# Spinal versus general anesthesia for patients undergoing outpatient total knee arthroplasty: a national propensity matched analysis of early postoperative outcomes

**DOI:** 10.1186/s12871-021-01442-2

**Published:** 2021-09-15

**Authors:** Mark C. Kendall, Alexander D. Cohen, Stephanie Principe-Marrero, Peter Sidhom, Patricia Apruzzese, Gildasio De Oliveira

**Affiliations:** 1 Department of Anesthesiology, The Warren Alpert Medical School of Brown University, 593 Eddy Street, Davol #129, Providence, RI 02903 USA; 2 Department of Anesthesiology, Rhode Island Hospital, Providence, RI USA

**Keywords:** Outpatient total knee arthroplasty, Spinal anesthesia, General anesthesia, Postoperative outcomes, Knee surgery

## Abstract

**Background:**

A comparison of different anesthetic techniques to evaluate short term outcomes has yet to be performed for patients undergoing outpatient knee replacements. The aim of this investigation was to compare short term outcomes of spinal (SA) versus general anesthesia (GA) in patients undergoing outpatient total knee replacements.

**Methods:**

The ACS NSQIP datasets were queried to extract patients who underwent primary, elective, unilateral total knee arthroplasty (TKA) between 2005 and 2018 performed as an outpatient procedure. The primary outcome was a composite score of serious adverse events (SAE). The primary independent variable was the type of anesthesia (e.g., general vs. spinal).

**Results:**

A total of 353,970 patients who underwent TKA procedures were identified comprising of 6,339 primary, elective outpatient TKA procedures. Of these, 2,034 patients received GA and 3,540 received SA. A cohort of 1,962 patients who underwent outpatient TKA under GA were propensity matched for covariates with patients who underwent outpatient TKA under SA. SAE rates at 72 h after surgery were not greater in patients receiving GA compared to SA (0.92%, 0.66%, *P* = 0.369). In contrast, minor adverse events were greater in the GA group compared to SA (2.09%, 0.51%), *P* < 0.001. The rate of postoperative transfusion was greater in the patients receiving GA.

**Conclusions:**

The type of anesthetic technique, general or spinal anesthesia does not alter short term SAEs, readmissions and failure to rescue in patients undergoing outpatient TKR surgery. Recognizing the benefits of SA tailored to the anesthetic management may maximize the clinical benefits in this patient population.

## Background

The demand for total knee arthroplasty (TKA) is expected to increase exponentially by 2050, and healthcare systems are exploring strategies to meet this demand in a safe and cost-effective manner [1, 2]. This has resulted in significantly more TKA procedures being performed on an outpatient basis in selected patients [3]. The shift from inpatient to outpatient TKA has significant potential of cost savings for health care systems and government payers (e.g., Medicare) [4].

Postoperative adverse events in patients undergoing TKA may increase the probability of disability and affect the quality of recovery. Previous investigations have reported the adverse events and serious adverse events ranging from 1 month to several years in duration [5–7]. Patients in the ambulatory setting cannot rely on hospital support (e.g., nurses, intravenous medications) to manage their postoperative recovery and are expected to provide self-care after surgery [8]. The type of anesthetic plan, general versus spinal anesthesia has been shown to influence the postoperative outcomes in patients undergoing TKA surgery.

The purpose of this study is to compare the association between spinal versus general anesthesia on early postoperative outcomes in patients undergoing outpatient TKAs using the most up-to-date sample from the American College of Surgeons National Surgical Quality Improvement Program (ACS-NSQIP) database. We hypothesized that patients undergoing outpatient TKA under spinal anesthesia would have a lower rate of serious postoperative complications when compared to patients receiving general anesthesia. In addition, we sought to compare readmission rates between the two anesthesia techniques.

## Methods

The study was performed in accordance with the ethical standards of the Declaration of Helsinki (1964) and its subsequent amendments. This study was performed under an exempt status granted by the Institutional Review Board (IRB#1647940). The IRB determined that the study qualified for exemption under 45 CFR 46.101(b). The exemption was granted because the study involved a retrospective review of existing data recorded in such a manner that subjects cannot be identified, directly or through identifiers linked to the subjects. Clinical information of the subjects was obtained for the years between 2005 and 2018 from the American College of Surgeons (ACS) National Surgical Quality Improvement Program (NSQIP) database. The study is reported following the STROBE guidelines for reporting observational studies [9].

The ACS-NSQIP database is a national prospective database that compiles voluntarily reported data from over 680 institutions in the United States. For example, over 1 million cases were submitted as part of the 2017 and 2018 update to the NSQIP database. Data is collected on over 300 variables that include preoperative risk factors, intraoperative variables and post-operative outcomes including complications up to 30 days after surgical procedures [10]. Data collection has been previously described in detail [10, 11]. In brief, data are collected in 8-day cycles, with the first 40 procedures in the cycle included in the dataset. The most commonly performed procedures are capped at 5 within each cycle to increase procedure heterogeneity. Trained clinical nurses assigned at each site collect data for 30 days postoperatively using isolated telephone interviews and operative and clinical notes. Interrater reliability audits of selected participating sites help ensure the collected data are of the highest quality possible. The combined results of inter-rater reliability audits completed to date revealed an overall inter-rater disagreement rate of approximately 1.8% for all assessed program variables [10, 11].

De-identified patient information is freely available to all institutional members who comply with the ACS NSQIP Data Use Agreement. The Data Use Agreement implements the protections afforded by the Health Insurance Portability and Accountability Act of 1996 and the ACS NSQIP Hospital Participation Agreement. The ACS NSQIP and the hospitals participating in this program are the sources of the data used in this study; however, these entities have not verified and are not responsible for the statistical validity of the data analysis or the conclusions derived by the authors [12, 13].

The 2005 through 2018 NSQIP Participant Use Data Files were queried to extract all patients scheduled. Patients who underwent primary, elective, unilateral TKA were identified using the Current Procedural Terminology (CPT) code 27,447. Cases involving trauma, fracture, neoplasms, infectious diseases, or patients under 18 were excluded. Patients who qualified for the study under these criteria were then further stratified to an outpatient TKA cohort, defined as length of stay (LOS) of 0 days [13].

### Outcomes variables and analysis

Preoperative demographic variables such as age, sex, body mass index, functional status, American Society of Anesthesiologists physical status (ASA PS) classification, smoking status, preoperative hematocrit, hypertension, diabetes, congestive heart failure, bleeding disorder and chronic obstructive pulmonary disease were compared between the two cohorts. Surgical duration was also compared between the cohorts. The primary independent variable was the type of anesthesia performed (general anesthesia or spinal anesthesia).

Postoperative events were grouped into serious adverse events (SAE), minor adverse events (MAE), and any adverse events (AE) following the same classification of prior studies on patients undergoing TKA [14, 15]. The primary outcome was serious adverse events defined as a composite that includes: (1) a return to operating room, (2) wound related infection, (3) thromboembolic event, (4) renal failure, (5) myocardial infarction, (6) cardiac arrest requiring cardiopulmonary resuscitation, (7) stroke or cerebrovascular accident, (8) on ventilator > 48 h, (9) unplanned intubation, (10) sepsis/septic shock, and (10) death. Minor adverse events included: (1) blood transfusion, (2) pneumonia, (3) wound dehiscence, (3) urinary tract infection and (4) renal insufficiency.

### Statistical analysis

Due to the observational (non-randomized) nature of this data, propensity score matching was used to minimize the effects of confounding when assessing differences in patient demographics between outpatient TKA procedures performed with spinal anesthesia and general anesthesia. In this study, the probability for undergoing an outpatient TKA procedure with general anesthesia (propensity score) was calculated for each patient based on age, sex, body mass index, ASA PS classification, functional status before surgery, smoking status, preoperative hematocrit, hypertension, diabetes, congestive heart failure, bleeding disorder, COPD, and surgical duration [16]. General anesthesia patients were one-to-one matched without replacement to a spinal anesthesia patient with the nearest propensity score, using a caliper of 0.10. If such a match was not available, the patient was eliminated. With this methodology, 1,962 general anesthesia patients were matched with patients who received spinal anesthesia.

Prior to matching, pre-operative demographics were compared using unpaired Student’s t test for continuous variables, and chi-square test for binary and categorical variables. Pre-operative demographics were compared in the matched cohorts using paired t-tests for continuous variables, McNemar’s Test for binary variables, and Bowker’s Symmetry test for categorical variables [17].

After propensity score matching, differences in outcome rates of the matched cohorts were assessed using McNemar’s test for matched data. Relative risks were calculated, as were risk differences. The rates of events between general anesthesia and spinal anesthesia cohorts were compared at 72 h postoperative (events that occurred at any time in the 72 h postoperative period). To account for multiple endpoint testing, an adjusted p-value was calculated to correct for False Discovery Rate (FDR).

All statistical analyses were conducted with the use of SAS software version 9.4 (SAS Institute Inc., Cary, North Carolina).

## Results

**Fig. 1:**
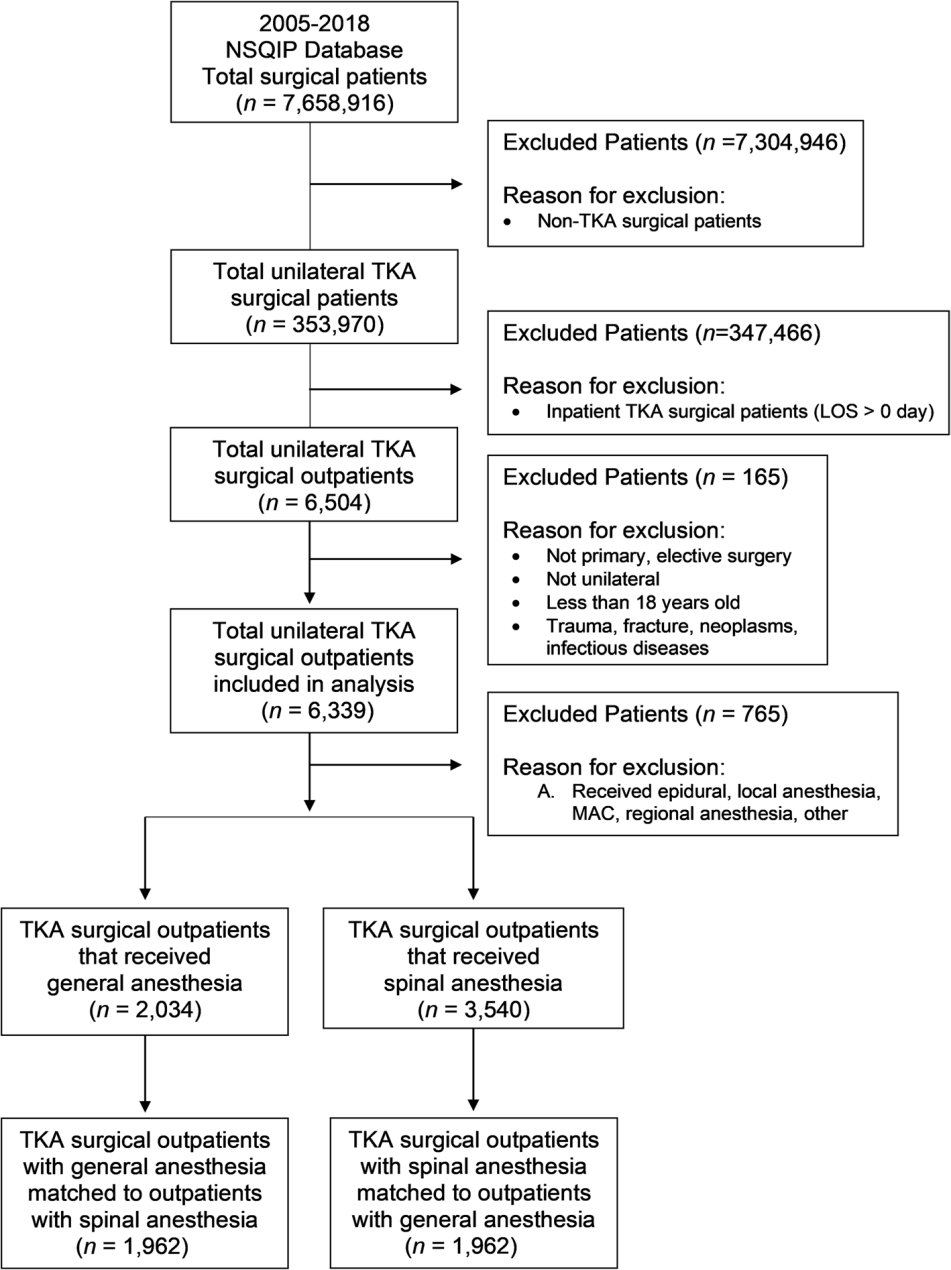
Flow diagram of included and excluded patients. NSQIP = National Surgical Quality Improvement; TKA = Total Knee Arthroplasty. Of these, 2,034 patients had surgery with general anesthesia and 3,540 had surgery with spinal anesthesia. A cohort of 1,962 patients who underwent TKA under general anesthesia were propensity matched with 1,962 patients who underwent TKA under spinal anesthesia. Patients in the outpatient group who had general anesthesia were younger (64.4 vs. 66.3, *P* < 0.001), and more likely to have a greater body mass index (32.6 vs 31.1) (*P* < 0.001) (Table 1). Covariates were well balanced between the propensity matched cohorts, absolute standard mean difference < 0.1 for all covariates (Table 2)

A total of 353,970 patients undergoing unilateral TKA were included in the NSQIP database for 2005–2018. A total of 6,504 patients underwent outpatient TKA and 5,574 were eligible after exclusion criteria (Fig. 1).

**Table 1:** Demographics of Patients Undergoing Unilateral Total Knee Arthroplasty before Propensity Matching Analysis

	**All Patients** **(n = 5,574)**	**Spinal Anesthesia** **(n = 3,540)**	**General Anesthesia** **(n = 2,034)**	**Difference** **(95% CI)**	P Value*
Age, (y), mean ± SD	65.61 ± 9.34	66.33 ± 8.68	64.36 ± 10.28	1.97 (1.44, 2.50)	< .001
Sex, % (n)					
Male	45.9 (2557/5574)	45.8 (1621/3540)	46.0 (936/2034)	-0.23 (-2.94, 2.49)	0.87
Female	54.1 (3017/5574)	54.2 (1919/3540)	53.9 (1098/2034)	0.23 (-2.49, 2.94)	
BMI, kg/m^2^, Mean ± SD	31.67 ± 6.03	31.12 ± 5.74	32.64 ± 6.38	-1.52 (-1.86, -1.19)	< .001
Functional status before surgery, % (n)					
Independent	99.6 (5553/5574)	99.7 (3530/3540)	99.5 (2023/2034)	0.26 (-0.11, 0.62)	0.13
Partially dependent	0.38 (21/5574)	0.28 (10/3540)	0.54 (11/2034)	-0.26 (-0.62, 0.11)	
Totally dependent	0.00 (0/5574)	0.00 (0/3540)	0.00 (0/2034)	0.00 (-, -)	
ASA PS, % (n)					
ASA PS 1 or 2	61.70 (3439/5574)	64.41 (2280/3540)	56.98 (1159/2034)	7.43 (4.76, 10.09)	< .001
ASA PS 3 or 4	38.30 (2135/5574)	35.59 (1260/3540)	43.02 (875/2034)	-7.43 (-10.09, -4.76)	
Smoker, % (n)					
No	93.02 (5185/5574)	94.07 (3330/3540)	91.20 (1855/2034)	2.87 (1.41, 4.32)	< .001
Yes	6.98 (389/5574)	5.93 (210/3540)	8.80 (179/2034)	-2.87 (-4.32, -1.41)	
Pre-op Hematocrit, Mean ± SD	41.75 ± 3.88	41.89 ± 3.69	41.50 ± 4.18	0.39 (0.17, 0.62]	< .001
Surgical Duration, min, Mean ± SD	83.02 ± 29.42	79.65 ± 23.57	88.88 ± 36.77	-9.23 (-11.00,-7.45)	< .001
Hypertension, % (n)	57.05 (3180/5574)	55.34 (1959/3540)	60.03 (1221/2034)	-4.69 (-7.38, -2.00)	< .001
Diabetes, % (n)	14.46 (806/5574)	13.33 (472/3540)	16.42 (334/2034)	-3.09 (-5.05, -1.13)	0.002
Congestive Heart Failure, % (n)	0.07 (4/5574)	0.06 (2/3540)	0.10 (2/2034)	-0.04 (-0.20, 0.12)	0.57
Bleeding disorder, % (n)	1.38 (77/5574)	1.07 (38/3540)	1.92 (39/2034)	-0.84 (-1.53, -0.16)	0.009
COPD, % (n)	2.15 (120/5574)	1.75 (62/3540)	2.85 (58/2034)	-1.10 (-1.94, -0.26)	0.006

χ2 test was used for binary and categorical variables, Student t-test was used for continuous variables

ASA PS = American Society of Anesthesiologists physical status classification system, BMI = body mass index, COPD = chronic obstructive pulmonary disease, SD = standard deviation

**Table 2:** Demographics of Patients Undergoing Unilateral Total Knee Arthroplasty after Propensity Matching Analysis

	**Spinal Anesthesia** **(n = 1,962)**	**General Anesthesia** **(n = 1,962)**	**Standardized Difference**	P Value*
Age, (y), mean ± SD	64.38 ± 8.70	64.62 ± 10.18	-0.02	0.38
Sex, % (n)				0.60
Male	46.53 (913/1962)	45.72 (897/1962)	0.01	
Female	53.47 (1049/1962)	54.28 (1065/1962)	-0.01	
BMI, kg/m^2^, Mean ± SD	32.31 ± 6.15	32.43 ± 6.23	-0.01	
Functional status before surgery, % (n)				0.83
Independent	99.49 (1952/1962)	99.44 (1951/1962)	0.007	
Partially dependent	0.51 (10/1962)	0.56 (11/1962)	-0.007	
Totally dependent	0.00 (0/1962)	0.00 (0/1962)		
ASA PS, % (n)				
ASA PS 1 or 2	59.07 (1159/1962)	57.65 (1131/1962)	0.02	0.36
ASA PS 3 or 4	40.93 (803/1962)	42.35 (831/1962)	-0.02	
Smoker, % (n)				
No	91.13 (1788/1962)	91.74 (1800/1962)	-0.02	0.47
Yes	8.87 (174/1962)	8.26 (162/1962)	0.02	
Pre-op Hematocrit, Mean ± SD	41.76 ± 3.68	41.60 ± 4	0.04	0.17
Surgical Duration, min, Mean ± SD	85.50 ± 26.64	86.28 ± 30.96	-0.02	0.24
Hypertension, % (n)	58.92 (1156/1962)	59.53 (1168/1962)	-0.01	0.69
Diabetes, % (n)	15.80 (310/1962)	15.75 (309/1962)	0.001	0.96
Congestive Heart Failure, % (n)	0.05 (1/1962)	0.05 (1/1962)	0.00	1.00
Bleeding disorder, % (n)	1.53 (30/1962)	1.63 (32/1962)	-0.008	0.79
COPD, % (n)	2.34 (46/1962)	2.60 (51/1962)	-0.01	0.61

^*^McNemar's test was used for binary variables, Bowker’s symmetry test for categorical variables, Paired t-test was used for continuous variables. ASA PS = American Society of Anesthesiologists physical status classification system, BMI = body mass index, COPD chronic obstructive pulmonary disease, SD = standard deviation

**Fig. 2:**
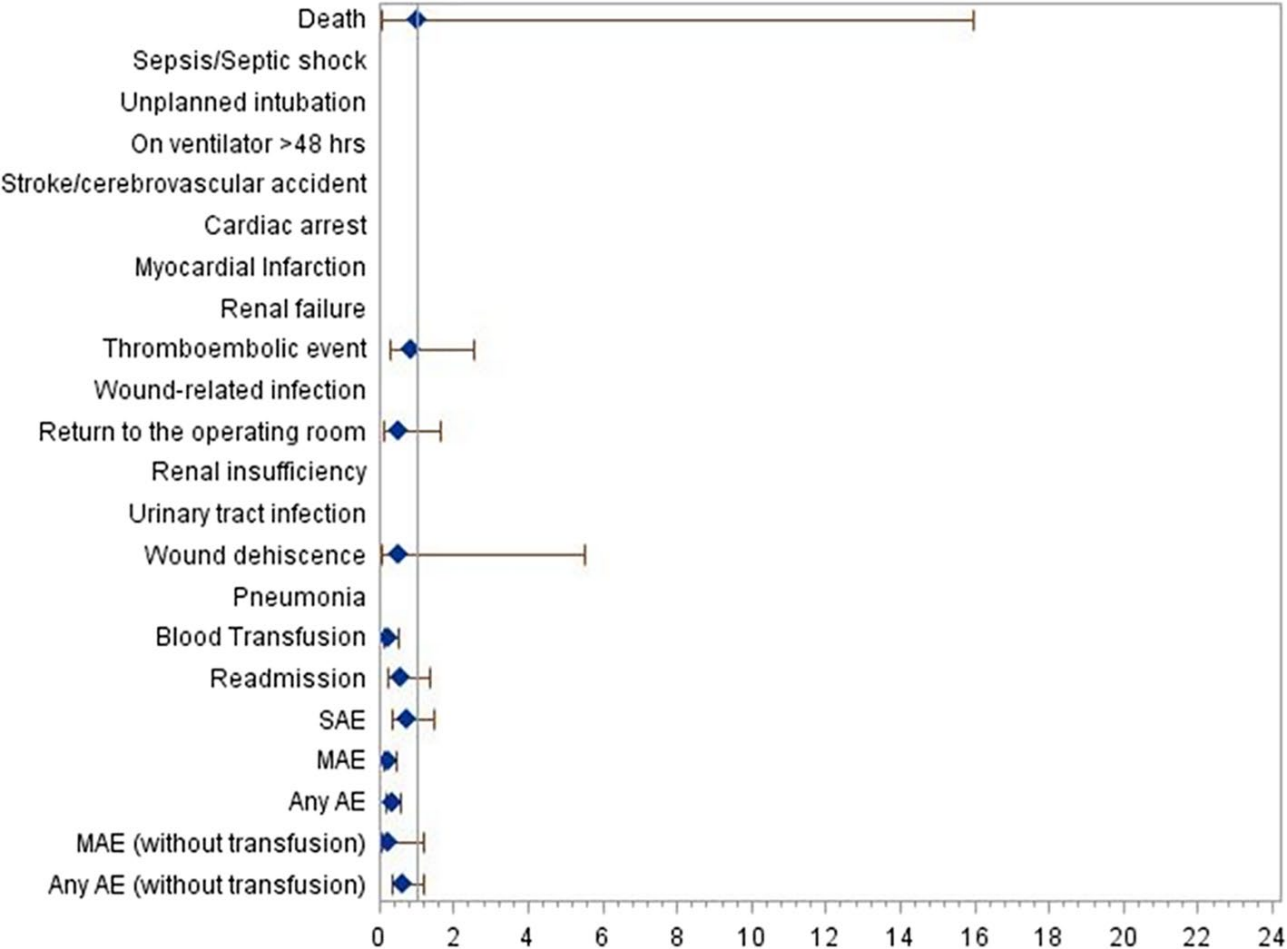
Forest Plots illustrating the relative risks of adverse events in outpatients undergoing total knee arthroplasty comparing general anesthesia to spinal anesthesia. Abbreviations: SSI = surgical site infection; VTE = venous thromboembolism; Diamonds represent the point estimate for relative risk; line represents 95% confidence intervals

Serious adverse events rates at 72 h were not greater in patients receiving general anesthesia compared to spinal anesthesia (0.92% vs. 0.66%, *P* = 0.369). In contrast, minor adverse events at 72 h after surgery were greater in the general anesthesia group compared to spinal anesthesia (2.09% vs. 0.51%), *P* < 0.001. In addition, the incidence of any adverse events at 72 h was also greater in the general anesthesia group (2.91% vs. 1.02%), *P* < 0.001. Specific rates for each adverse event comparing patients who had TKAs under general anesthesia to patients who had TKAs under spinal anesthesia are presented on Table 3. Specifically, the rate of blood transfusions 72 h after surgery were lower in the spinal anesthesia group compared to the general anesthesia, false discovery adjusted rate of *P* = 0.0004. Relative risks for each adverse event comparing general anesthesia to spinal anesthesia in TKA outpatients are presented in Fig. 2.

**Table 3:** Matched Comparisons and Relative Risk of Adverse Event Rates that Occurred 72 h After Surgery in Spinal vs General Anesthesia for Outpatient Total Knee Arthroplasty

	**Spinal** **Anesthesia** **(n = 1,962)**	**General** **Anesthesia** **(n = 1,962)**	**Risk Difference** **(95% CI)**	**Relative Risk** **(95% CI)**	P Value*	**FDR** **P Value†**
Death	1 (0.05)	1 (0.05)	0.00 (-0.14, 0.14)	1.00 (0.06, 15.98)	1.00	1.00
Sepsis/Septic shock	0 (0.00)	0 (0.00)	0.00 (-, -)	-	-	-
Unplanned intubation	2 (0.10)	0 (0.00)	0.10 (-0.04, 0.24)	-	0.15	0.33
On ventilator > 48 h	0 (0.00)	1 (0.05)	-0.05 (-0.15, 0.05)	-	0.31	0.37
Stroke/cerebrovascular accident	0 (0.00)	1 (0.05)	-0.05 (-0.15, 0.05)	-	0.31	0.37
Cardiac arrest	0 (0.00)	3 (0.15)	-0.15 (-0.33, 0.02)	-	0.08	0.22
Myocardial Infarction	0 (0.00)	3 (0.15)	-0.15 (-0.33, 0.02)	-	0.08	0.22
Renal failure	0 (0.00)	0 (0.00)	0.00 (-, -)	-	-	-
Thromboembolic event	6 (0.31)	7 (0.36)	-0.05 (-0.41, 0.31)	0.86 (0.29, 2.55)	0.78	0.82
Wound-related infection	1 (0.05)	0 (0.00)	0.05 (-0.05, 0.15)	-	0.31	0.37
Return to the operating room	4 (0.20)	8 (0.41)	-0.20 (-0.55, 0.14)	0.50 (0.15, 1.66)	0.24	0.37
Renal insufficiency	0 (0.00)	1 (0.05)	-0.05 (-0.15, 0.05)	-	0.31	0.37
Urinary tract infection	1 (0.05)	0 (0.00)	0.05 (-0.05, 0.15)	-	0.31	0.37
Wound dehiscence	1 (0.05)	2 (0.10)	-0.05 (-0.22, 0.12)	0.50 (0.05, 5.51)	0.56	0.63
Pneumonia	0 (0.00)	5 (0.25)	-0.25 (-0.48, -0.03)	-	0.02	0.12
Blood Transfusion	8 (0.41)	33 (1.68)	-1.27 (-1.91, -0.64)	0.24 (0.11, 0.52)	< .001	0.0004
Readmission	9 (0.46)	15 (0.78)	-0.32 (-0.81, 0.18)	0.59 (0.26, 1.35)	0.22	0.37
SAE	13 (0.66)	18 (0.92)	-0.25 (-0.81,0.30)	0.72 (0.35, 1.47)	0.36	-
MAE	10 (0.51)	41 (2.09)	-1.58 (-2.29, -0.87)	0.24 (0.12, 0.49)	< .001	0.0002
Any AE	20 (1.02)	57 (2.91)	-1.89 (-2.75, -1.02)	0.35 (0.21, 0.58)	< .001	0.0002
MAE (without transfusion)	2 (0.10)	8 (0.41)	-0.31 (-0.62, 0.01)	0.25 (0.05, 1.18)	0.058	0.21
Any AE (without transfusion)	15 (0.76)	24 (1.22)	-0.46 (-1.08,0.16)	0.63 (0.33, 1.19)	0.15	0.33

Data presented as n (%) unless otherwise stated. *P values were calculated using McNemar’s test for matched data. AE = adverse events, CI = confidence interval, SAE = serious adverse events, MAE = minor adverse events. FDR = false discovery rate used for multiple comparisons.

Of note, readmission rates did not significantly differ between the groups. Failure to rescue in the matched cohort also did not differ from general and spinal anesthesia, 1/20 vs 1/57, *P* = 0.45.

## Discussion

The most important finding of the current investigation was the lack of a difference in early serious adverse events when spinal anesthesia and general anesthesia were used for outpatient TKA. In contrast, the composite rate of early minor adverse events and any adverse events were greater in patients receiving general anesthesia compared to spinal anesthesia for outpatient TKA. Specifically, the need of postoperative blood transfusion was greater in patients receiving general anesthesia compared to regional anesthesia. Taken together, our results suggest that spinal anesthesia provides selective clinical advantages in the early recovery period when compared to general anesthesia for patients undergoing outpatient TKA.

Previous studies have compared general anesthesia to spinal anesthesia with conflicting results in patients undergoing TKAs in the inpatient setting. For example, Warren et al*.* detected a decreased rate of complications in patients undergoing inpatient TKA with spinal anesthesia compared to those receiving general anesthesia [18]. In contrast, Nakamura et al*.* reported an increased rate of venous thromboembolism in patients receiving spinal anesthesia for TKA [19]. Nevertheless, as far as we are aware, no study has evaluated the impact between the type of anesthesia technique on patient outcomes after outpatient TKAs.

Our results are clinically important given the current shift of practice towards the performance of total knee replacement in the outpatient setting [20]. Given the current financial incentives and economic pressures to reduce costs, it is expected that the number of outpatient total knee replacement procedures are expected to grow substantially over the following years [21]. To the best of our knowledge, this is the first study to compare the safety of neuraxial versus general anesthetic techniques in the outpatient setting for total knee replacement.

Prior studies examining inpatient TKAs have resulted in conflicting results regarding the effect of spinal anesthesia in reducing transfusion rates when compared to general anesthesia. Rashiq et al*.* did not detect a benefit of spinal anesthesia to reduce transfusion after inpatient TKAs [22]. In contrast, Wei et al.detected a benefit of spinal anesthesia to reduce transfusion after inpatient TKAs [23]. Our results are critical since patients who have been discharged and need blood transfusions have less access to care (e.g., regular vital signs monitoring and blood tests) to recognize the need for the transfusion in the outpatient setting.

It was also interesting to note the selection process for the patients undergoing outpatient TKA who received spinal anesthesia. In the original cohort, patients in the outpatient setting who received spinal anesthesia were older and had lower BMIs than patients who received general anesthesia. We used propensity score matching to adjust for the covariate imbalances in our analysis and obtained a well-adjusted cohort (e.g., standard mean difference < 0.1 for all covariates). It is possible that clinical practitioners wanted to avoid general anesthesia in older patients due to the potential risk of postoperative delirium and/or cognitive decline [24]. Patients with greater BMI may provide challenges to the performance of spinal anesthesia and this may explain the greater choice of general anesthesia to this population [25].

We did not detect a greater rate of readmissions and/or failure to rescue in the general anesthesia group compared to the spinal anesthesia group. This collaborates the lack of difference in serious adverse events between the study groups. In the case of blood transfusions, it is possible that patients came to the emergency room to receive a blood transfusion but were not admitted. Unfortunately, the NSQIP database does not track emergency room visits and we could not confirm or refute that assumption.

Our study can only be interpreted within the context of its limitations. With a large, multi-institutional database such as the ACS-NSQIP, there are well published limitations including the possibility of clerical error, differences in inter-rater reliability across institutions and only 30-day postoperative follow-up window. In order to avoid overfitting of our models, we did not include all variables collected at NSQIP. Lastly, due to limitations on the database, we cannot assess potential drug usage variations in the anesthesia techniques that could potentially alter the outcome. For example, it is possible that some patients in the general anesthesia group received sugammadex to reverse neuromuscular blockage while others did not receive it.

## Conclusions

In summary, the type of anesthesia technique does not alter short term serious adverse events, readmissions, and failure to rescue in patients undergoing outpatient total knee replacement. In contrast, patients who received general anesthesia reported a greater rate of minor and any adverse events compared to patients who received spinal anesthesia. Specifically, the rate of blood transfusions was reduced in patients who received spinal anesthesia compared to general anesthesia in the early postoperative period. Clinicians should recognize the benefits of spinal anesthesia in patients undergoing outpatient knee replacements and tailor the anesthetic plan to maximize clinical benefits.

## Data Availability

Data is available from the authors upon reasonable request and with permission of ACS NSQIP. The URL is https://www.facs.org/Quality-Programs/ACS-NSQIP/participant-use. The American College of Surgeons National Surgical Quality Improvement Program and the hospitals participating in the ACS NSQIP are the source of the data used herein; they have not verified and are not responsible for the statistical validity of the data analysis or the conclusions derived by the authors. The data that support the findings of this study are available from ACS NSQIP but restrictions apply to the availability of these data, which were used under license for the current study, and so are not publicly available.

## Abbreviations

ACS: American College of Surgeons
ASA PS: American Society of Anesthesiologists physical status classification
CI: Confidence interval
CPT: Current procedural terminology
IRB: Institutional review board
LOS: Length of stay
NSQIP: National surgical quality improvement program database
STROBE: Strengthening the reporting of observational studies in epidemiology.
TKA: Total knee arthroplasty
